# Synthesis and crystal structure of poly[[bis­(aqua-κ*O*)tetra­kis­(μ-4,4′-bi­pyridine-κ^2^ *N*:*N*′)hexa­kis­(3-chloro­benzoato)-κ^5^ *O*;κ^2^ *O*:*O*′-tricobalt(II)] methanol disolvate]

**DOI:** 10.1107/S2056989022000731

**Published:** 2022-02-01

**Authors:** Phakamat Promwit, Kittipong Chainok, Nanthawat Wannarit

**Affiliations:** a Department of Chemistry, Faculty of Science and Technology, Thammasat University, Klong Luang, Pathum Thani 12121, Thailand; b Thammasat University Research Unit in Multifunctional Crystalline Materials and Applications (TU-MCMA), Faculty of Science and Technology, Thammasat University, Klong Luang, Pathum Thani 12121, Thailand

**Keywords:** cobalt(II), one-dimensional coord­in­ation polymer, ladder-chain structure, 4,4′-bi­pyridine, 3-Clbenz, crystal structure

## Abstract

A novel ladder-chain cobalt(II) coordination polymer, {[Co_3_(C_7_H_4_ClO_2_)_6_(C_10_H_8_N_2_)_4_(H_2_O)_2_]·2CH_3_OH}_*n*_, was synthesized and characterized. The structure contains two Co^II^ centres with different octa­hedral environments, [Co(1)N_3_O_3_] and [Co(2)N_2_O_4_]. The *O*-donating 3-chloro­benzoate anions (3-Clbenz) act as the terminal ligands, while the *N*-donating 4,4′-bipy mol­ecules play the role of linkers. The Co(1) ions are linked by 4,4′-bipy mol­ecules into linear chains. Two such chains are joined by [Co(2)(3-Clbenz)_2_(H_2_O)_2_] units *via* two 4,4′-bipy bridging ligands, thus forming the ladder-chain structure. The crystal packing of the title compound is stabilized by supra­molecular inter­actions, such as hydrogen bonding, π–π and halogen⋯π contacts, giving a three-dimensional framework. The spectroscopic and thermal properties of the title compound have also been investigated.

## Abstract 2

The synthesis, crystal structure and properties of a novel ladder-chain Co^II^ coordination polymer constructed from 4,4′-bi­pyridine (4,4′-bipy) and *m*-chloro­benzoate (3-Clbenz), {[Co_3_(4,4′-bipy)_4_(3-Clbenz)_6_(H_2_O)_2_]·2CH_3_OH}_*n*_, are reported.

## Chemical context

The exploration and synthesis of new one-dimensional coordination polymers based on transition metals and mixed *N*- and *O*-donating ligands such as 4,4′-bi­pyridine (4,4′-bipy) and benzoate derivatives have been intensively developed (Kaes *et al.*, 2000 ▸; Saelim *et al.*, 2020 ▸; Topor *et al.*, 2021 ▸). The substituent groups at the benzoate ligands play an important role not only for electron densities on the aromatic ring, but also for flexible supra­molecular inter­actions, resulting in various bulk physical properties, such as CO_2_ adsorption (Takahashi *et al.*, 2014 ▸, 2015 ▸), photoluminescence (Lin, 2015 ▸) and conductivity (Islam *et al.*, 2019 ▸). Among the reported compounds, the majority contain mixed 4,4′-bipy and *para*-substituted benzoate deriv­atives, but there is a limited number of examples containing *meta*-substituent benzoate ligands (Fang & Nie, 2011 ▸; Kar *et al.*, 2011 ▸; Xin-Jian *et al.*, 2013 ▸; Lin, 2015 ▸). We have therefore tried to expand investigations in this area by using various *meta*-substituted benzoate ligands containing hy­droxy, nitro and halogen substituents. During this study, we employed 3-chloro­benzoate (3-Clbenz), which is expected to support crystal structures *via* π–π and halogen⋯π inter­molecular inter­actions, together with the 4,4′-bipy organic linker and have synthesized the new Co^II^ coordination polymer {[Co_3_(4,4′-bipy)_4_(3-Clbenz)_6_(H_2_O)_2_]·2CH_3_OH}_*n*_, which has an inter­esting one-dimensional ladder-chain structure. This report describes the synthesis, crystal structure, spectroscopic and thermal properties of the title compound. 
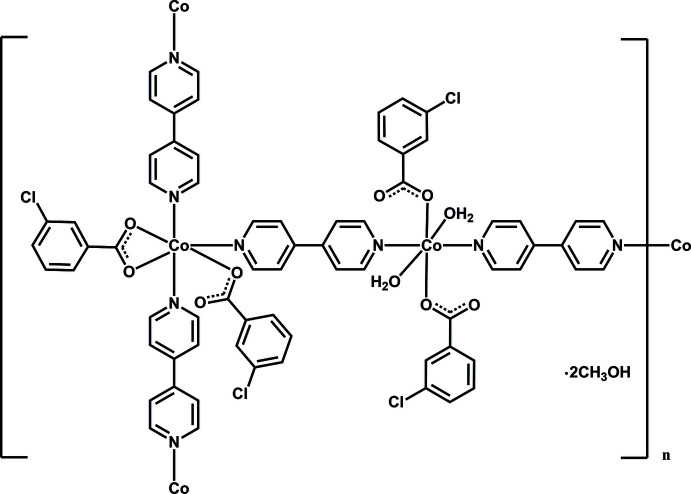


## Structural commentary

**Figure 1:**
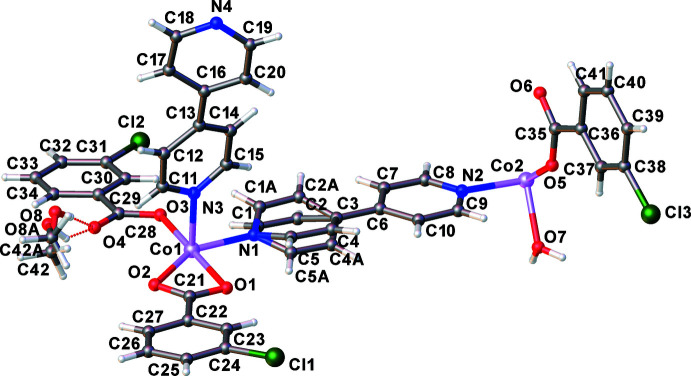
Asymmetric unit of the title compound with the atom labelling. Displacement ellipsoids are drawn at the 50% probability level.

**Figure 2:**
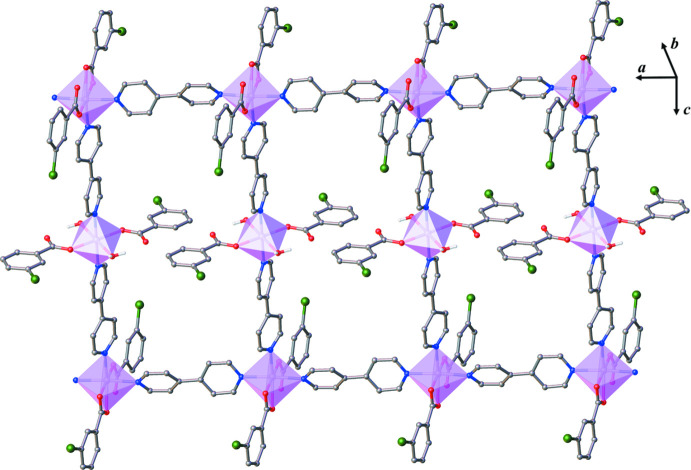
View of the ladder-chain structure along the *a-*axis direction. The hydrogen atoms located at carbon atoms and methanol solvate mol­ecules are omitted for clarity.

The asymmetric unit of the title compound comprises two Co^2+^ ions, three 3-Clbenz anions, two 4,4′-bipy mol­ecules, one coordinated water mol­ecule and one methanol solvate mol­ecule as shown in Fig. 1 ▸. One of the Co^2+^ ions (containing Co2), is situated at an inversion centre. One pyridine ring (C1–C5) and the methanol solvate mol­ecule are disordered over two sets of sites with occupancies of 0.584 (19):0.416 (19) and 0.72 (3):0.28 (3), respectively. Both Co^2+^ ions are six-coordinated and have octa­hedral environments. The Co1 ion is coordinated by three nitro­gen atoms from three 4,4′-bipy bridging ligands and three oxygen atoms from the carboxyl­ate groups of one monodentate and one bidentate 3-Clbenz ligands, providing a distorted octa­hedral geometry with angles O2—Co1—O1, O2—Co1—O3 and O2—Co1—N1 of 59.88 (6), 119.93 (7) and 148.87 (7)°, respectively. The Co2 ion is coordinated by two nitro­gen atoms from two 4,4′-bipy bridging ligands and four oxygen atoms from two monodentate 3-Clbenz ligands and two coordinated water mol­ecules. The angles in its environment deviate from ideal values no more than by 3.5°. There is an intra­molecular hydrogen bond in the coordination environment of Co2 between the aqua and 3-Clbenz ligands (see Table 1 ▸). The Co1 ions are connected by the 4,4′-bipy linkers into linear chains along the *a-*axis direction, and adjacent chains are linked *via* the Co2 ions by the 4,4′-bipy ligands, thus forming the ladder-chain structure shown in Fig. 2 ▸.

## Supra­molecular features

**Figure 3:**
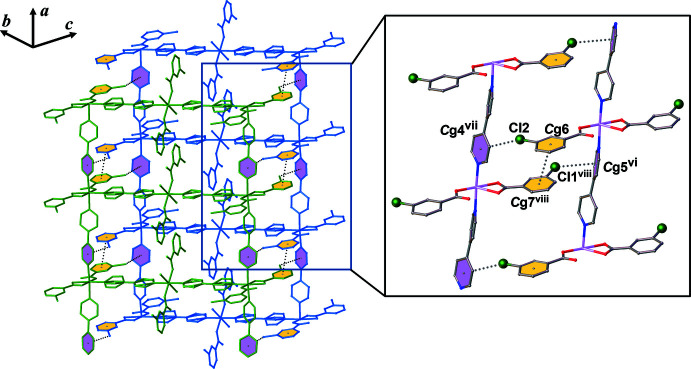
Views of the inter­molecular π–π and Cl⋯π inter­actions between adjacent ladder chains [symmetry codes: (vi) *x*, −1 + *y*, *z*; (vii) −1 + *x*, 1 + *y*, *z*; (viii) 1 + *x*, −1 + *y*, *z*].

**Figure 4:**
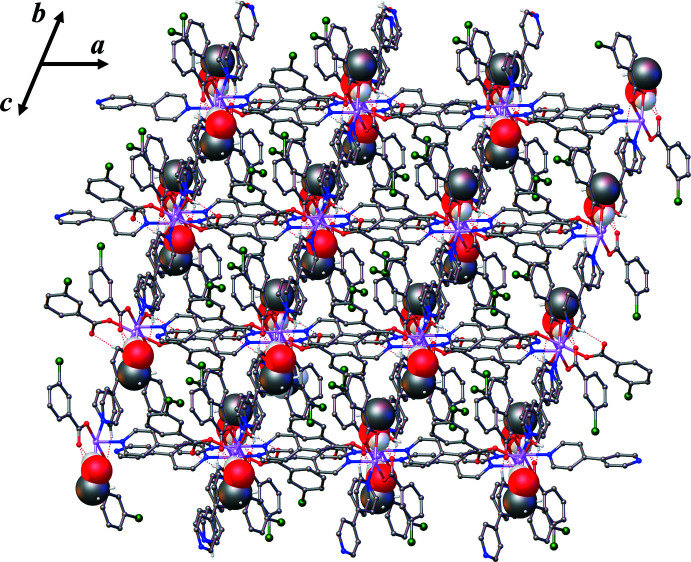
Packing diagram of the title compound viewed along the [011] direction**.** C-bound hydrogen atoms are omitted for clarity. Methanol solvate mol­ecules are indicated by larger balls.

The crystal packing is stabilized by inter­molecular inter­actions such as hydrogen bonds (classical O—H⋯O and non-classical C—H⋯O and C—H⋯N), aromatic π–π and Cl⋯π inter­actions (see Table 1 ▸, Figs. 3 ▸ and 4 ▸). The solvate methanol mol­ecule forms hydrogen bonds to the non-coordinated O4 atom of the 3-Clbenz ligand at Co1 as an H-atom donor and to the coordinated water mol­ecule at Co2 as an H-atom acceptor (see Figs. S1–S3 in the supporting information). Aromatic π–π inter­actions involving the phenyl rings of two 3-Clbenz ligands have an inter­centroid *Cg*6⋯*Cg*7 (1 + *x*, −1 + *y*, *z*) separation of 3.917 (2) Å (Fig. 3 ▸) (*Cg*6 and *Cg*7 are the centroids of the C22–C27 and C29–C34 rings, respectively). There are also halogen⋯π inter­actions between the 3-Clbenz ligands and the pyridine rings of 4,4′-bipy ligands with C24—Cl1⋯*Cg*5 (*x*, −1 + *y*, *z*) = 3.5833 (14) Å and C31—Cl2⋯*Cg*4 (−1 + *x*, 1 + *y*, *z*) = 3.7558 (15) Å (Fig. 3 ▸) (*Cg*4 and *Cg*5 are the centroids of the N3/C11–C15 and N4/C16–C20 rings, respectively). These inter­actions stabilize the structure, leading to a three-dimensional supra­molecular framework (Fig. 4 ▸).

## Spectroscopic characterization

The FT–IR spectrum of the title compound (Fig. S4) has a characteristic broad peak centred at 3330 cm^−1^ assigned to the O—H stretching vibrations of coordinated water mol­ecules and the methanol solvate. The strong and sharp peaks at about 1608 and 1382 cm^−1^ are attributed to the asymmetric and symmetric COO^−^ stretching vibration of the monodentate 3-Clbenz ligands, and the peaks appearing at about 1557 and 1488 cm^−1^ are attributed to the asymmetric and symmetric COO^−^ stretching vibration of the chelating 3-Clbenz ligand (Xin-Jian *et al.*, 2013 ▸). The strong superimposed bands appearing at 1557 and 1488 cm^−1^ could be assigned to the C=C/C=N stretching vibration of the aromatic rings of the 3-Clbenz and 4,4′-bipy ligands. The medium-strong peaks in the region of 760 and 731 cm^−1^ are assigned to C—Cl vibration and C—H bending vibration of the 3-Clbenz ligands. In addition, the medium-strong peak at 1219 cm^−1^ is assigned to the weak C—N stretching vibration (Xin-Jian *et al.*, 2013 ▸) and the bands between 1016 and 1145 cm^−1^ are assignable to the pyridine ring-breathing modes (Dey *et al.*, 2011 ▸) of the 4,4′-bipy ligands. The characteristic C—H out-of-plane and in-plane deformation bands for pyridine rings are observed at 808 and 631 cm^−1^, and are shifted to a higher frequency as compared to the values observed for the free ligand (805 and 607 cm^−1^), suggesting coordinated 4,4′-bipy ligands (Seidel *et al.*, 2011 ▸). The solid-state electronic spectrum (Fig. S5) of the title compound shows *d–d* transitions with two broad bands at 489 and 1099 nm, assigned to the ν_3_: ^4^ *T*_1*g*_ → ^4^ *T*_1*g*_(*P*) and ν_1_: ^4^ *T*_1g_ → ^4^ *T*_2*g*_ transitions, respectively (Fu *et al.*, 2007 ▸; Piromchom *et al.*, 2014 ▸). The results correspond to the typical *d–d* transitions for Co^II^ in a distorted octa­hedral geometry, as confirmed by the X-ray structure.

## PXRD and thermal analysis

The PXRD patterns (Fig. S6) of the title compound used to check the phase purity show good accordance with its simulated PXRD pattern generated from the single-crystal X-ray diffraction data, confirming that the title compound has high phase purity. The TGA curve (Fig. S7) shows the thermal stability of the title compound below 325°C. The first complex step with a weight loss of 29.57% (calculated 30.88%) was found in the temperature range from 100 to 325°C, which was attributed to the loss of methanol mol­ecule of crystallization, two coordinated water and three 3-Clbenz mol­ecules. Then, the structure starts to collapse with a weight loss of 49.24% (calculated 49.44%) in the temperature range from 325–685°C that can be attributed to the removal of three remaining 3-Clbenz and three remaining 4,4′-bipy mol­ecules. After that, the residual product is assumed to be CoO.

## Database survey

To the best of our knowledge, only two transition-metal-based coordination polymers structurally related to the title compound, namely [Co_3_(dca)_2_(nic)_4_(H_2_O)_8_]·2H_2_O (CSD refcode XOGLOU; Kutasi *et al.*, 2002 ▸) and [Cu_3_(dca)_2_(nic)_4_(H_2_O)_8_]·2H_2_O (KAPMOE; Madalan *et al.*, 2005 ▸) (dca = dicyanamide and nic = 3-pyridine­carboxyl­ate) are reported in the literature. These compounds are isostructural to each other and differ only by the kind of transition metal.

## Synthesis and crystallization

A methano­lic solution (5 ml) of 4,4′-bipy (0.4586 g, 3 mmol) was added to a solution of Co(NO_3_)_2_·6H_2_O (0.2910 g, 1 mmol) in 10 mL of MeOH/H_2_O (*v*:*v* = 8:2) solution. After stirring for 30 min, a methano­lic solution (5 mL) of *m*-chloro­benzoic acid (0.3131 g, 2 mmol) was added slowly, and the mixture was stirred continuously at room temperature for 15 minutes. The resulting clear red solution was allowed to evaporate slowly in air. After 4 days, red rod-shaped crystals suitable for single-crystal X-ray diffraction were obtained. Yield 115.2 mg (32.6% based on Co^II^ salt). Analysis calculated for C_84_H_68_Cl_6_Co_3_N_8_O_16_: C, 54.98; H, 3.74; N, 6.11%. Found: C, 53.28; H, 3.60; N, 6.50%. IR (KBr, cm^−1^): 3330(*w*), 2348(*w*), 1608(*s*), 1557(*s*), 1488(*w*), 1415(*s*), 1382(*s*), 1263(*w*), 1219(*m*), 1145(*w*), 1068(*m*), 1031(*w*), 1010(*w*), 817(*m*), 808(*m*), 760(*m*), 731(*m*), 674(*w*), 657(*w*), 631(*m*), 574(*w*), 499(*w*), 439(*w*).

## Refinement

Crystal data, data collection and structure refinement details are summarized in Table 2 ▸. All C-bound hydrogen atoms were positioned geometrically and refined as riding, with C—H = 0.96 Å for methyl groups [*U*_iso_(H) = 1.5 *U*_eq_(C)], C—H = 0.93 Å for aromatic [*U*_iso_(H) = 1.2 *U*_eq_(C)]. The oxygen-bound hydrogen atom of methanol was positioned with O—H = 0.82 Å [*U*_iso_(H) = 1.5*U*_eq_(O)], and the OH group was allowed to rotate (AFIX 147). Hydrogen atoms of the coordinated water mol­ecule were located in the differential electron density map and refined with the O—H distance contrained to 0.84 Å.

## Supplementary Material

Crystal structure: contains datablock(s) I. DOI: 10.1107/S2056989022000731/yk2163sup1.cif

Structure factors: contains datablock(s) I. DOI: 10.1107/S2056989022000731/yk2163Isup2.hkl

Supporting data of crystal structure, characterization and physical properties. DOI: 10.1107/S2056989022000731/yk2163sup4.pdf

CCDC reference: 2143539

Additional supporting information:  crystallographic information; 3D view; checkCIF report

## Figures and Tables

**Table 1:** Hydrogen-bond geometry (Å, °)

*D*—H⋯*A*	*D*—H	H⋯*A*	*D*⋯*A*	*D*—H⋯*A*
O7—H7*A*⋯O6^i^	0.85 (2)	1.84 (2)	2.648 (3)	158 (3)
O7—H7*B*⋯O8^ii^	0.85 (2)	1.93 (2)	2.777 (10)	177 (3)
O7—H7*B*⋯O8*A* ^ii^	0.85 (2)	1.88 (4)	2.72 (3)	168 (3)
O8—H8*A*⋯O4	0.82	1.90	2.708 (10)	169
O8*A*—H8*AA*⋯O4	0.82	2.04	2.67 (3)	133
C1—H1⋯O3	0.93	2.59	3.102 (7)	115
C5—H5⋯O1	0.93	2.48	3.057 (9)	121
C1*A*—H1*A*⋯O3	0.93	2.52	3.088 (9)	120
C5*A*—H5*A*⋯O1	0.93	2.33	2.991 (12)	128
C9—H9⋯O5	0.93	2.71	3.189 (3)	113
C11—H11⋯O2	0.93	2.57	3.084 (3)	115
C15—H15⋯N1	0.93	2.60	3.198 (4)	123
C26—H26⋯O6^iii^	0.93	2.60	3.524 (4)	176

Symmetry codes: (i) 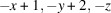 ; (ii) 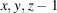 ; (iii) 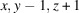 .

**Table 2:** Experimental details

Crystal data	
Chemical formula	[Co_3_(C_7_H_4_ClO_2_)_6_(C_10_H_8_N_2_)_4_(H_2_O)_2_]·2CH_4_O
*M*_r_	1834.95
Crystal system, space group	Triclinic, *P* 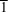
Temperature (K)	296
*a*, *b*, *c* (Å)	11.388 (2), 11.868 (2), 18.055 (3)
α, β, γ (°)	79.516 (6), 79.088 (6), 62.148 (6)
*V* (Å^3^)	2106.3 (7)
*Z*	1
Radiation type	Mo *K*α
μ (mm^−1^)	0.84
Crystal size (mm)	0.43 × 0.32 × 0.26

Data collection	
Diffractometer	Bruker D8 QUEST CMOS PHOTON II
Absorption correction	Multi-scan (*SADABS*; Bruker, 2016 ▸)
*T*_min_, *T*_max_	0.684, 0.745
No. of measured, independent and observed [*I* > 2σ(*I*)] reflections	78843, 8352, 6158
*R*_int_	0.077
(sin θ/λ)_max_ (Å^−1^)	0.621

Refinement	
*R*[*F* ^2^ > 2σ(*F* ^2^)], *wR*(*F* ^2^), *S*	0.037, 0.093, 1.02
No. of reflections	8352
No. of parameters	598
No. of restraints	43
H-atom treatment	H atoms treated by a mixture of independent and constrained refinement
Δρ_max_, Δρ_min_ (e Å^−3^)	0.39, −0.36

Computer programs: *APEX3* and *SAINT* (Bruker, 2016 ▸), *SHELXT* (Sheldrick, 2015*a* ▸), *SHELXL* (Sheldrick, 2015*b* ▸) and *OLEX2* (Dolomanov *et al.*, 2009 ▸).

## supplementary crystallographic information

### Crystal data

**Table:**

[Co_3_(C_7_H_4_ClO_2_)_6_(C_10_H_8_N_2_)_4_(H_2_O)_2_]·2CH_4_O	*Z* = 1
*M**_r_* = 1834.95	*F*(000) = 939
Triclinic, *P*1	*D*_x_ = 1.447 Mg m^−^^3^
*a* = 11.388 (2) Å	Mo *K*α radiation, λ = 0.71073 Å
*b* = 11.868 (2) Å	Cell parameters from 9884 reflections
*c* = 18.055 (3) Å	θ = 2.9–25.9°
α = 79.516 (6)°	µ = 0.84 mm^−^^1^
β = 79.088 (6)°	*T* = 296 K
γ = 62.148 (6)°	Block, red
*V* = 2106.3 (7) Å^3^	0.43 × 0.32 × 0.26 mm

### Data collection

**Table:**

Bruker D8 QUEST CMOS PHOTON II diffractometer	8352 independent reflections
Radiation source: sealed x-ray tube, Mo	6158 reflections with *I* > 2σ(*I*)
Graphite monochromator	*R*_int_ = 0.077
Detector resolution: 7.39 pixels mm^-1^	θ_max_ = 26.2°, θ_min_ = 2.9°
ω and φ scans	*h* = −14→14
Absorption correction: multi-scan (SADABS; Bruker, 2016)	*k* = −14→14
*T*_min_ = 0.684, *T*_max_ = 0.745	*l* = −22→22
78843 measured reflections	

### Refinement

**Table:**

Refinement on *F*^2^	Hydrogen site location: mixed
Least-squares matrix: full	H atoms treated by a mixture of independent and constrained refinement
*R*[*F*^2^ > 2σ(*F*^2^)] = 0.037	*w* = 1/[σ^2^(*F*_o_^2^) + (0.0352*P*)^2^ + 1.2109*P*] where *P* = (*F*_o_^2^ + 2*F*_c_^2^)/3
*wR*(*F*^2^) = 0.093	(Δ/σ)_max_ = 0.001
*S* = 1.02	Δρ_max_ = 0.39 e Å^−^^3^
8352 reflections	Δρ_min_ = −0.36 e Å^−^^3^
598 parameters	Extinction correction: SHELXL (Sheldrick, 2015b), Fc^*^=kFc[1+0.001xFc^2^λ^3^/sin(2θ)]^-1/4^
43 restraints	Extinction coefficient: 0.0034 (6)

### Special details

**Table:**

Geometry. All esds (except the esd in the dihedral angle between two l.s. planes) are estimated using the full covariance matrix. The cell esds are taken into account individually in the estimation of esds in distances, angles and torsion angles; correlations between esds in cell parameters are only used when they are defined by crystal symmetry. An approximate (isotropic) treatment of cell esds is used for estimating esds involving l.s. planes.

### Fractional atomic coordinates and isotropic or equivalent isotropic displacement parameters (Å^2^)

**Table:**

	*x*	*y*	*z*	*U*_iso_*/*U*_eq_	Occ. (<1)
Co1	0.50698 (3)	0.68260 (3)	0.63422 (2)	0.03063 (10)	
Co2	0.500000	1.000000	0.000000	0.03999 (13)	
Cl1	0.99436 (12)	0.00343 (9)	0.59982 (7)	0.1103 (4)	
Cl2	0.05072 (9)	1.34860 (8)	0.61650 (5)	0.0794 (3)	
Cl3	1.09130 (10)	0.45840 (8)	−0.12014 (5)	0.0856 (3)	
O1	0.63032 (18)	0.48413 (16)	0.60490 (10)	0.0500 (4)	
O2	0.56402 (17)	0.52368 (16)	0.72324 (10)	0.0471 (4)	
O3	0.39489 (17)	0.86355 (16)	0.66137 (10)	0.0500 (4)	
O4	0.3946 (2)	0.80593 (19)	0.78514 (12)	0.0682 (6)	
O5	0.70423 (17)	0.88325 (17)	−0.02018 (9)	0.0489 (4)	
O6	0.79423 (19)	1.0110 (2)	−0.00999 (12)	0.0637 (5)	
O7	0.4670 (2)	0.83959 (19)	−0.00306 (11)	0.0554 (5)	
H7A	0.3828 (18)	0.870 (3)	−0.0018 (18)	0.070 (10)*	
H7B	0.504 (3)	0.789 (3)	−0.0370 (14)	0.074 (11)*	
O8	0.5835 (9)	0.6811 (6)	0.8812 (6)	0.070 (2)	0.72 (3)
H8A	0.534267	0.713202	0.847635	0.105*	0.72 (3)
O8A	0.552 (4)	0.669 (3)	0.893 (2)	0.118 (10)	0.28 (3)
H8AA	0.513149	0.667322	0.860315	0.177*	0.28 (3)
N1	0.50401 (17)	0.74863 (17)	0.51524 (10)	0.0320 (4)	
N2	0.5003 (2)	0.94739 (19)	0.12052 (10)	0.0436 (5)	
N3	0.68941 (18)	0.69508 (18)	0.63878 (11)	0.0373 (4)	
N4	1.32607 (18)	0.66423 (18)	0.63798 (11)	0.0378 (4)	
C1	0.4041 (7)	0.8562 (8)	0.4894 (4)	0.0409 (18)	0.584 (19)
H1	0.336133	0.906039	0.524106	0.049*	0.584 (19)
C2	0.3979 (7)	0.8961 (8)	0.4134 (3)	0.0422 (17)	0.584 (19)
H2	0.324386	0.970146	0.397732	0.051*	0.584 (19)
C4	0.6000 (9)	0.7124 (8)	0.3873 (5)	0.0385 (19)	0.584 (19)
H4	0.668235	0.658787	0.354224	0.046*	0.584 (19)
C5	0.5979 (9)	0.6781 (9)	0.4639 (4)	0.0368 (19)	0.584 (19)
H5	0.666182	0.600802	0.481176	0.044*	0.584 (19)
C1A	0.4507 (18)	0.8730 (9)	0.4867 (5)	0.052 (3)	0.416 (19)
H1A	0.411651	0.934175	0.520990	0.062*	0.416 (19)
C2A	0.4498 (19)	0.9156 (8)	0.4113 (4)	0.056 (4)	0.416 (19)
H2A	0.415636	1.002838	0.395275	0.067*	0.416 (19)
C4A	0.5608 (15)	0.7006 (10)	0.3868 (6)	0.038 (3)	0.416 (19)
H4A	0.602070	0.637977	0.353428	0.045*	0.416 (19)
C5A	0.5608 (15)	0.6653 (12)	0.4634 (6)	0.038 (3)	0.416 (19)
H5A	0.602681	0.578372	0.480289	0.046*	0.416 (19)
C3	0.5007 (2)	0.8269 (2)	0.35885 (12)	0.0355 (5)	
C6	0.5011 (2)	0.8683 (2)	0.27628 (12)	0.0374 (5)	
C7	0.4220 (3)	0.9934 (2)	0.24883 (13)	0.0470 (6)	
H7	0.367277	1.053757	0.282340	0.056*	
C8	0.4246 (3)	1.0285 (2)	0.17172 (14)	0.0504 (7)	
H8	0.370780	1.113144	0.154564	0.060*	
C9	0.5766 (3)	0.8273 (3)	0.14692 (14)	0.0582 (8)	
H9	0.630829	0.769104	0.112168	0.070*	
C10	0.5794 (3)	0.7848 (3)	0.22277 (14)	0.0563 (7)	
H10	0.634247	0.699523	0.238210	0.068*	
C11	0.7468 (2)	0.6564 (2)	0.70265 (13)	0.0415 (6)	
H11	0.702007	0.632002	0.746159	0.050*	
C12	0.8689 (2)	0.6506 (2)	0.70775 (13)	0.0401 (5)	
H12	0.904054	0.623321	0.753865	0.048*	
C13	0.9389 (2)	0.6852 (2)	0.64437 (13)	0.0350 (5)	
C14	0.8759 (3)	0.7325 (3)	0.57973 (15)	0.0589 (8)	
H14	0.915922	0.762362	0.536209	0.071*	
C15	0.7537 (3)	0.7356 (3)	0.57951 (15)	0.0599 (8)	
H15	0.713650	0.768076	0.534968	0.072*	
C16	1.0742 (2)	0.6734 (2)	0.64405 (13)	0.0341 (5)	
C17	1.1168 (2)	0.6868 (2)	0.70762 (13)	0.0405 (6)	
H17	1.061642	0.698869	0.753556	0.049*	
C18	1.2415 (2)	0.6820 (2)	0.70222 (14)	0.0433 (6)	
H18	1.268121	0.691561	0.745340	0.052*	
C19	1.2866 (2)	0.6468 (2)	0.57783 (14)	0.0433 (6)	
H19	1.344921	0.631851	0.533042	0.052*	
C20	1.1646 (2)	0.6498 (2)	0.57855 (14)	0.0439 (6)	
H20	1.142397	0.635907	0.535167	0.053*	
C21	0.6305 (2)	0.4468 (2)	0.67475 (15)	0.0407 (6)	
C22	0.7160 (2)	0.3079 (2)	0.69985 (14)	0.0425 (6)	
C23	0.8002 (3)	0.2268 (2)	0.64613 (16)	0.0516 (7)	
H23	0.798960	0.256315	0.594778	0.062*	
C24	0.8861 (3)	0.1018 (3)	0.66913 (18)	0.0633 (8)	
C25	0.8881 (4)	0.0552 (3)	0.7441 (2)	0.0755 (10)	
H25	0.946344	−0.029321	0.758825	0.091*	
C26	0.8032 (4)	0.1350 (3)	0.79735 (18)	0.0734 (9)	
H26	0.803440	0.103845	0.848487	0.088*	
C27	0.7169 (3)	0.2615 (3)	0.77596 (16)	0.0566 (7)	
H27	0.659947	0.314862	0.812564	0.068*	
C28	0.3553 (2)	0.8863 (2)	0.73000 (15)	0.0433 (6)	
C29	0.2493 (2)	1.0208 (2)	0.74272 (14)	0.0409 (6)	
C30	0.2048 (2)	1.1118 (2)	0.68156 (14)	0.0443 (6)	
H30	0.241121	1.091367	0.632359	0.053*	
C31	0.1062 (3)	1.2331 (2)	0.69406 (15)	0.0497 (6)	
C32	0.0488 (3)	1.2658 (3)	0.76603 (18)	0.0640 (8)	
H32	−0.019254	1.347439	0.773607	0.077*	
C33	0.0945 (3)	1.1749 (3)	0.82652 (18)	0.0721 (9)	
H33	0.057886	1.195925	0.875601	0.086*	
C34	0.1941 (3)	1.0526 (3)	0.81560 (15)	0.0582 (7)	
H34	0.223845	0.992042	0.857068	0.070*	
C35	0.8013 (3)	0.9088 (3)	−0.02400 (13)	0.0460 (6)	
C36	0.9385 (3)	0.8046 (3)	−0.04884 (13)	0.0477 (6)	
C37	0.9508 (3)	0.6923 (3)	−0.06973 (14)	0.0502 (6)	
H37	0.875389	0.680489	−0.068912	0.060*	
C38	1.0771 (3)	0.5980 (3)	−0.09184 (15)	0.0590 (8)	
C39	1.1901 (3)	0.6134 (4)	−0.09343 (19)	0.0752 (10)	
H39	1.274104	0.549159	−0.108321	0.090*	
C40	1.1773 (3)	0.7249 (4)	−0.0728 (2)	0.0822 (10)	
H40	1.253102	0.736253	−0.073692	0.099*	
C41	1.0521 (3)	0.8204 (3)	−0.05062 (17)	0.0663 (8)	
H41	1.044221	0.895601	−0.036837	0.080*	
C42	0.601 (2)	0.5527 (8)	0.9055 (6)	0.098 (4)	0.72 (3)
H42A	0.690754	0.492804	0.889708	0.148*	0.72 (3)
H42B	0.539583	0.537897	0.883146	0.148*	0.72 (3)
H42C	0.582855	0.541560	0.959750	0.148*	0.72 (3)
C42A	0.661 (3)	0.544 (2)	0.909 (2)	0.112 (9)	0.28 (3)
H42D	0.743858	0.542832	0.884862	0.168*	0.28 (3)
H42E	0.646889	0.480899	0.890648	0.168*	0.28 (3)
H42F	0.664034	0.524534	0.963145	0.168*	0.28 (3)

### Atomic displacement parameters (Å^2^)

**Table:**

	*U*^11^	*U*^22^	*U*^33^	*U*^12^	*U*^13^	*U*^23^
Co1	0.02389 (15)	0.03470 (17)	0.03223 (17)	−0.01262 (13)	−0.00512 (12)	−0.00071 (12)
Co2	0.0419 (3)	0.0426 (3)	0.0269 (2)	−0.0121 (2)	−0.00361 (19)	−0.00320 (19)
Cl1	0.1260 (9)	0.0567 (5)	0.1072 (8)	−0.0018 (5)	−0.0107 (7)	−0.0273 (5)
Cl2	0.0859 (6)	0.0508 (4)	0.0776 (6)	−0.0133 (4)	−0.0140 (4)	0.0039 (4)
Cl3	0.0938 (6)	0.0531 (5)	0.0815 (6)	−0.0070 (4)	−0.0091 (5)	−0.0155 (4)
O1	0.0551 (11)	0.0391 (10)	0.0466 (11)	−0.0144 (8)	−0.0147 (8)	0.0069 (8)
O2	0.0441 (10)	0.0418 (10)	0.0529 (11)	−0.0168 (8)	−0.0090 (8)	−0.0025 (8)
O3	0.0432 (10)	0.0454 (10)	0.0546 (11)	−0.0130 (8)	0.0027 (8)	−0.0172 (8)
O4	0.0709 (14)	0.0511 (12)	0.0674 (14)	−0.0140 (10)	−0.0191 (11)	0.0024 (10)
O5	0.0422 (10)	0.0506 (11)	0.0456 (10)	−0.0136 (8)	−0.0041 (8)	−0.0071 (8)
O6	0.0567 (12)	0.0623 (13)	0.0704 (14)	−0.0230 (10)	0.0014 (10)	−0.0232 (11)
O7	0.0581 (14)	0.0516 (12)	0.0523 (12)	−0.0193 (10)	−0.0037 (10)	−0.0137 (9)
O8	0.081 (4)	0.064 (3)	0.051 (3)	−0.018 (2)	−0.011 (2)	−0.0108 (18)
O8A	0.136 (14)	0.118 (12)	0.091 (15)	−0.030 (9)	−0.047 (12)	−0.031 (9)
N1	0.0288 (10)	0.0362 (10)	0.0299 (10)	−0.0132 (8)	−0.0064 (8)	−0.0021 (8)
N2	0.0467 (12)	0.0432 (12)	0.0300 (10)	−0.0117 (10)	−0.0034 (9)	−0.0034 (9)
N3	0.0281 (10)	0.0439 (11)	0.0415 (11)	−0.0184 (9)	−0.0064 (8)	0.0002 (9)
N4	0.0296 (10)	0.0452 (11)	0.0416 (11)	−0.0198 (9)	−0.0066 (8)	−0.0010 (9)
C1	0.033 (3)	0.043 (3)	0.033 (3)	−0.009 (2)	−0.001 (2)	−0.001 (2)
C2	0.035 (3)	0.041 (3)	0.036 (3)	−0.007 (2)	−0.003 (2)	0.000 (2)
C4	0.035 (4)	0.041 (3)	0.033 (3)	−0.014 (3)	0.000 (2)	−0.003 (2)
C5	0.032 (4)	0.036 (3)	0.036 (3)	−0.011 (3)	−0.002 (2)	−0.001 (2)
C1A	0.079 (9)	0.034 (4)	0.034 (4)	−0.019 (5)	−0.002 (5)	−0.006 (3)
C2A	0.087 (9)	0.031 (4)	0.030 (4)	−0.014 (5)	−0.004 (5)	0.004 (3)
C4A	0.046 (7)	0.031 (4)	0.033 (4)	−0.016 (4)	0.004 (4)	−0.007 (3)
C5A	0.047 (7)	0.028 (4)	0.037 (4)	−0.017 (4)	0.001 (4)	0.003 (3)
C3	0.0372 (12)	0.0389 (13)	0.0301 (12)	−0.0165 (10)	−0.0051 (9)	−0.0034 (10)
C6	0.0401 (13)	0.0395 (13)	0.0301 (12)	−0.0153 (11)	−0.0057 (10)	−0.0034 (10)
C7	0.0592 (16)	0.0401 (14)	0.0299 (12)	−0.0131 (12)	−0.0017 (11)	−0.0059 (10)
C8	0.0598 (17)	0.0391 (14)	0.0357 (13)	−0.0099 (12)	−0.0057 (12)	−0.0002 (11)
C9	0.0654 (18)	0.0500 (16)	0.0313 (13)	−0.0024 (14)	−0.0022 (12)	−0.0089 (12)
C10	0.0663 (18)	0.0410 (15)	0.0354 (14)	−0.0018 (13)	−0.0082 (12)	−0.0033 (11)
C11	0.0324 (12)	0.0595 (16)	0.0356 (13)	−0.0246 (12)	0.0005 (10)	−0.0055 (11)
C12	0.0346 (12)	0.0540 (15)	0.0359 (13)	−0.0227 (11)	−0.0075 (10)	−0.0034 (11)
C13	0.0297 (11)	0.0378 (13)	0.0404 (13)	−0.0171 (10)	−0.0066 (10)	−0.0025 (10)
C14	0.0499 (16)	0.096 (2)	0.0457 (15)	−0.0507 (17)	−0.0148 (12)	0.0187 (15)
C15	0.0509 (16)	0.097 (2)	0.0464 (15)	−0.0487 (17)	−0.0236 (13)	0.0225 (15)
C16	0.0305 (12)	0.0360 (12)	0.0393 (13)	−0.0181 (10)	−0.0061 (10)	−0.0009 (10)
C17	0.0338 (12)	0.0567 (15)	0.0358 (13)	−0.0260 (12)	−0.0021 (10)	−0.0018 (11)
C18	0.0403 (13)	0.0622 (16)	0.0359 (13)	−0.0297 (13)	−0.0110 (11)	0.0011 (11)
C19	0.0344 (13)	0.0591 (16)	0.0427 (14)	−0.0257 (12)	0.0014 (10)	−0.0135 (12)
C20	0.0379 (13)	0.0619 (16)	0.0418 (14)	−0.0279 (12)	−0.0028 (11)	−0.0154 (12)
C21	0.0364 (13)	0.0386 (13)	0.0512 (16)	−0.0207 (11)	−0.0122 (11)	0.0037 (12)
C22	0.0454 (14)	0.0383 (13)	0.0486 (15)	−0.0221 (12)	−0.0159 (12)	0.0048 (11)
C23	0.0588 (17)	0.0414 (15)	0.0524 (16)	−0.0206 (13)	−0.0148 (13)	0.0030 (12)
C24	0.069 (2)	0.0385 (15)	0.073 (2)	−0.0139 (14)	−0.0186 (16)	−0.0039 (14)
C25	0.095 (3)	0.0388 (16)	0.083 (2)	−0.0189 (17)	−0.037 (2)	0.0115 (17)
C26	0.104 (3)	0.0550 (19)	0.0584 (19)	−0.0361 (19)	−0.0314 (19)	0.0229 (16)
C27	0.0692 (19)	0.0516 (17)	0.0501 (16)	−0.0285 (15)	−0.0152 (14)	0.0045 (13)
C28	0.0356 (13)	0.0472 (15)	0.0518 (16)	−0.0213 (11)	−0.0043 (12)	−0.0099 (13)
C29	0.0390 (13)	0.0431 (14)	0.0436 (14)	−0.0182 (11)	−0.0046 (11)	−0.0121 (11)
C30	0.0426 (14)	0.0451 (14)	0.0436 (14)	−0.0173 (12)	−0.0006 (11)	−0.0124 (11)
C31	0.0467 (15)	0.0429 (15)	0.0558 (16)	−0.0155 (12)	−0.0068 (12)	−0.0087 (12)
C32	0.0641 (19)	0.0477 (17)	0.068 (2)	−0.0122 (14)	0.0007 (15)	−0.0239 (15)
C33	0.082 (2)	0.068 (2)	0.0523 (18)	−0.0204 (18)	0.0089 (16)	−0.0305 (16)
C34	0.0671 (19)	0.0606 (18)	0.0422 (15)	−0.0228 (15)	−0.0057 (13)	−0.0117 (13)
C35	0.0473 (15)	0.0530 (16)	0.0273 (12)	−0.0147 (13)	−0.0036 (11)	−0.0027 (11)
C36	0.0445 (15)	0.0586 (17)	0.0299 (13)	−0.0164 (13)	−0.0016 (11)	−0.0025 (11)
C37	0.0510 (16)	0.0524 (16)	0.0375 (14)	−0.0160 (13)	−0.0064 (12)	−0.0008 (12)
C38	0.0604 (18)	0.0526 (17)	0.0397 (15)	−0.0056 (14)	−0.0045 (13)	−0.0056 (12)
C39	0.0452 (18)	0.085 (2)	0.066 (2)	−0.0058 (17)	0.0022 (15)	−0.0128 (18)
C40	0.0489 (19)	0.103 (3)	0.088 (3)	−0.0281 (19)	0.0047 (17)	−0.023 (2)
C41	0.0550 (18)	0.076 (2)	0.066 (2)	−0.0276 (16)	0.0037 (15)	−0.0194 (16)
C42	0.136 (10)	0.072 (4)	0.077 (4)	−0.035 (4)	−0.039 (5)	0.009 (3)
C42A	0.114 (12)	0.102 (11)	0.121 (15)	−0.044 (8)	−0.020 (11)	−0.022 (9)

### Geometric parameters (Å, º)

**Table:**

Co1—O1	2.2096 (18)	C9—H9	0.9300
Co1—O2	2.1703 (17)	C9—C10	1.372 (3)
Co1—O3	2.0166 (17)	C10—H10	0.9300
Co1—N1	2.1494 (18)	C11—H11	0.9300
Co1—N3	2.1683 (18)	C11—C12	1.380 (3)
Co1—N4^i^	2.1584 (18)	C12—H12	0.9300
Co2—O5^ii^	2.0770 (17)	C12—C13	1.380 (3)
Co2—O5	2.0770 (17)	C13—C14	1.376 (3)
Co2—O7^ii^	2.117 (2)	C13—C16	1.479 (3)
Co2—O7	2.117 (2)	C14—H14	0.9300
Co2—N2^ii^	2.1520 (19)	C14—C15	1.376 (3)
Co2—N2	2.1519 (19)	C15—H15	0.9300
Cl1—C24	1.747 (3)	C16—C17	1.388 (3)
Cl2—C31	1.751 (3)	C16—C20	1.388 (3)
Cl3—C38	1.749 (3)	C17—H17	0.9300
O1—C21	1.259 (3)	C17—C18	1.380 (3)
O2—C21	1.252 (3)	C18—H18	0.9300
O3—C28	1.268 (3)	C19—H19	0.9300
O4—C28	1.235 (3)	C19—C20	1.372 (3)
O5—C35	1.263 (3)	C20—H20	0.9300
O6—C35	1.247 (3)	C21—C22	1.505 (3)
O7—H7A	0.849 (17)	C22—C23	1.383 (4)
O7—H7B	0.848 (18)	C22—C27	1.385 (4)
O8—H8A	0.8200	C23—H23	0.9300
O8—C42	1.436 (9)	C23—C24	1.380 (4)
O8A—H8AA	0.8200	C24—C25	1.366 (4)
O8A—C42A	1.445 (17)	C25—H25	0.9300
N1—C1	1.330 (7)	C25—C26	1.371 (5)
N1—C5	1.332 (8)	C26—H26	0.9300
N1—C1A	1.348 (9)	C26—C27	1.387 (4)
N1—C5A	1.335 (10)	C27—H27	0.9300
N2—C8	1.331 (3)	C28—C29	1.511 (3)
N2—C9	1.329 (3)	C29—C30	1.382 (3)
N3—C11	1.334 (3)	C29—C34	1.384 (3)
N3—C15	1.325 (3)	C30—H30	0.9300
N4—C18	1.340 (3)	C30—C31	1.377 (3)
N4—C19	1.333 (3)	C31—C32	1.375 (4)
C1—H1	0.9300	C32—H32	0.9300
C1—C2	1.372 (7)	C32—C33	1.375 (4)
C2—H2	0.9300	C33—H33	0.9300
C2—C3	1.405 (6)	C33—C34	1.383 (4)
C4—H4	0.9300	C34—H34	0.9300
C4—C5	1.368 (8)	C35—C36	1.518 (4)
C4—C3	1.386 (8)	C36—C37	1.387 (4)
C5—H5	0.9300	C36—C41	1.386 (4)
C1A—H1A	0.9300	C37—H37	0.9300
C1A—C2A	1.364 (10)	C37—C38	1.387 (4)
C2A—H2A	0.9300	C38—C39	1.376 (5)
C2A—C3	1.388 (8)	C39—H39	0.9300
C4A—H4A	0.9300	C39—C40	1.375 (5)
C4A—C5A	1.372 (11)	C40—H40	0.9300
C4A—C3	1.366 (10)	C40—C41	1.384 (4)
C5A—H5A	0.9300	C41—H41	0.9300
C3—C6	1.482 (3)	C42—H42A	0.9600
C6—C7	1.383 (3)	C42—H42B	0.9600
C6—C10	1.381 (3)	C42—H42C	0.9600
C7—H7	0.9300	C42A—H42D	0.9600
C7—C8	1.377 (3)	C42A—H42E	0.9600
C8—H8	0.9300	C42A—H42F	0.9600
			
O2—Co1—O1	59.88 (6)	C11—C12—H12	120.0
O3—Co1—O1	179.75 (7)	C13—C12—C11	119.9 (2)
O3—Co1—O2	119.93 (7)	C13—C12—H12	120.0
O3—Co1—N1	91.08 (7)	C12—C13—C16	123.1 (2)
O3—Co1—N3	91.04 (7)	C14—C13—C12	116.3 (2)
O3—Co1—N4^i^	89.24 (7)	C14—C13—C16	120.6 (2)
N1—Co1—O1	89.12 (7)	C13—C14—H14	120.0
N1—Co1—O2	148.87 (7)	C15—C14—C13	120.0 (2)
N1—Co1—N3	93.25 (7)	C15—C14—H14	120.0
N1—Co1—N4^i^	90.81 (7)	N3—C15—C14	124.1 (2)
N3—Co1—O1	88.80 (7)	N3—C15—H15	117.9
N3—Co1—O2	89.10 (7)	C14—C15—H15	117.9
N4^i^—Co1—O1	90.91 (7)	C17—C16—C13	122.6 (2)
N4^i^—Co1—O2	87.24 (7)	C20—C16—C13	120.7 (2)
N4^i^—Co1—N3	175.93 (7)	C20—C16—C17	116.7 (2)
O5—Co2—O5^ii^	180.0	C16—C17—H17	120.2
O5^ii^—Co2—O7^ii^	88.68 (8)	C18—C17—C16	119.6 (2)
O5—Co2—O7^ii^	91.32 (8)	C18—C17—H17	120.2
O5—Co2—O7	88.68 (8)	N4—C18—C17	123.3 (2)
O5^ii^—Co2—O7	91.32 (8)	N4—C18—H18	118.4
O5—Co2—N2	91.93 (7)	C17—C18—H18	118.4
O5^ii^—Co2—N2^ii^	91.93 (7)	N4—C19—H19	118.3
O5^ii^—Co2—N2	88.07 (7)	N4—C19—C20	123.4 (2)
O5—Co2—N2^ii^	88.07 (7)	C20—C19—H19	118.3
O7^ii^—Co2—O7	180.00 (11)	C16—C20—H20	120.0
O7—Co2—N2^ii^	93.42 (8)	C19—C20—C16	120.0 (2)
O7^ii^—Co2—N2^ii^	86.58 (8)	C19—C20—H20	120.0
O7—Co2—N2	86.58 (8)	O1—C21—C22	119.0 (2)
O7^ii^—Co2—N2	93.42 (8)	O2—C21—O1	121.1 (2)
N2—Co2—N2^ii^	180.0	O2—C21—C22	119.8 (2)
C21—O1—Co1	88.42 (15)	C23—C22—C21	119.3 (2)
C21—O2—Co1	90.38 (14)	C23—C22—C27	119.4 (2)
C28—O3—Co1	120.99 (17)	C27—C22—C21	121.2 (2)
C35—O5—Co2	129.83 (17)	C22—C23—H23	120.2
Co2—O7—H7A	104 (2)	C24—C23—C22	119.7 (3)
Co2—O7—H7B	125 (2)	C24—C23—H23	120.2
H7A—O7—H7B	108 (3)	C23—C24—Cl1	118.4 (2)
C42—O8—H8A	109.5	C25—C24—Cl1	120.3 (2)
C42A—O8A—H8AA	109.5	C25—C24—C23	121.4 (3)
C1—N1—Co1	121.7 (3)	C24—C25—H25	120.5
C1—N1—C5	117.2 (5)	C24—C25—C26	119.0 (3)
C5—N1—Co1	120.9 (4)	C26—C25—H25	120.5
C1A—N1—Co1	124.4 (4)	C25—C26—H26	119.6
C5A—N1—Co1	120.6 (5)	C25—C26—C27	120.9 (3)
C5A—N1—C1A	115.0 (7)	C27—C26—H26	119.6
C8—N2—Co2	123.55 (16)	C22—C27—C26	119.7 (3)
C9—N2—Co2	119.64 (16)	C22—C27—H27	120.2
C9—N2—C8	116.8 (2)	C26—C27—H27	120.2
C11—N3—Co1	120.60 (15)	O3—C28—C29	116.0 (2)
C15—N3—Co1	123.58 (16)	O4—C28—O3	124.4 (2)
C15—N3—C11	115.8 (2)	O4—C28—C29	119.6 (2)
C18—N4—Co1^iii^	119.07 (15)	C30—C29—C28	120.2 (2)
C19—N4—Co1^iii^	123.78 (15)	C30—C29—C34	119.6 (2)
C19—N4—C18	116.89 (19)	C34—C29—C28	120.2 (2)
N1—C1—H1	118.9	C29—C30—H30	120.3
N1—C1—C2	122.3 (6)	C31—C30—C29	119.5 (2)
C2—C1—H1	118.9	C31—C30—H30	120.3
C1—C2—H2	119.5	C30—C31—Cl2	119.4 (2)
C1—C2—C3	121.1 (5)	C32—C31—Cl2	119.0 (2)
C3—C2—H2	119.5	C32—C31—C30	121.7 (3)
C5—C4—H4	120.0	C31—C32—H32	120.8
C5—C4—C3	120.1 (8)	C33—C32—C31	118.4 (3)
C3—C4—H4	120.0	C33—C32—H32	120.8
N1—C5—C4	124.0 (8)	C32—C33—H33	119.5
N1—C5—H5	118.0	C32—C33—C34	121.1 (3)
C4—C5—H5	118.0	C34—C33—H33	119.5
N1—C1A—H1A	117.6	C29—C34—H34	120.1
N1—C1A—C2A	124.7 (8)	C33—C34—C29	119.8 (3)
C2A—C1A—H1A	117.6	C33—C34—H34	120.1
C1A—C2A—H2A	120.5	O5—C35—C36	116.0 (2)
C1A—C2A—C3	119.1 (8)	O6—C35—O5	126.2 (2)
C3—C2A—H2A	120.5	O6—C35—C36	117.8 (2)
C5A—C4A—H4A	119.7	C37—C36—C35	120.0 (2)
C3—C4A—H4A	119.7	C41—C36—C35	120.5 (3)
C3—C4A—C5A	120.7 (10)	C41—C36—C37	119.5 (3)
N1—C5A—C4A	123.7 (11)	C36—C37—H37	120.4
N1—C5A—H5A	118.2	C38—C37—C36	119.1 (3)
C4A—C5A—H5A	118.2	C38—C37—H37	120.4
C2—C3—C6	123.2 (3)	C37—C38—Cl3	118.7 (3)
C4—C3—C2	115.1 (5)	C39—C38—Cl3	119.8 (2)
C4—C3—C6	121.6 (4)	C39—C38—C37	121.4 (3)
C2A—C3—C6	121.2 (4)	C38—C39—H39	120.4
C4A—C3—C2A	116.6 (6)	C40—C39—C38	119.3 (3)
C4A—C3—C6	121.9 (5)	C40—C39—H39	120.4
C7—C6—C3	121.4 (2)	C39—C40—H40	119.9
C10—C6—C3	122.2 (2)	C39—C40—C41	120.2 (3)
C10—C6—C7	116.5 (2)	C41—C40—H40	119.9
C6—C7—H7	120.1	C36—C41—H41	119.8
C8—C7—C6	119.8 (2)	C40—C41—C36	120.5 (3)
C8—C7—H7	120.1	C40—C41—H41	119.8
N2—C8—C7	123.4 (2)	O8—C42—H42A	109.5
N2—C8—H8	118.3	O8—C42—H42B	109.5
C7—C8—H8	118.3	O8—C42—H42C	109.5
N2—C9—H9	118.3	H42A—C42—H42B	109.5
N2—C9—C10	123.4 (2)	H42A—C42—H42C	109.5
C10—C9—H9	118.3	H42B—C42—H42C	109.5
C6—C10—H10	119.9	O8A—C42A—H42D	109.5
C9—C10—C6	120.2 (2)	O8A—C42A—H42E	109.5
C9—C10—H10	119.9	O8A—C42A—H42F	109.5
N3—C11—H11	118.2	H42D—C42A—H42E	109.5
N3—C11—C12	123.7 (2)	H42D—C42A—H42F	109.5
C12—C11—H11	118.2	H42E—C42A—H42F	109.5
			
Co1—O1—C21—O2	4.4 (2)	C5A—C4A—C3—C2A	4.7 (12)
Co1—O1—C21—C22	−173.47 (19)	C5A—C4A—C3—C6	178.9 (7)
Co1—O2—C21—O1	−4.5 (2)	C3—C4—C5—N1	0.3 (10)
Co1—O2—C21—C22	173.37 (19)	C3—C4A—C5A—N1	0.0 (14)
Co1—O3—C28—O4	10.1 (3)	C3—C6—C7—C8	−179.9 (2)
Co1—O3—C28—C29	−167.67 (15)	C3—C6—C10—C9	−180.0 (3)
Co1—N1—C1—C2	−176.8 (4)	C6—C7—C8—N2	0.1 (4)
Co1—N1—C5—C4	178.0 (5)	C7—C6—C10—C9	0.1 (4)
Co1—N1—C1A—C2A	179.2 (7)	C8—N2—C9—C10	0.5 (4)
Co1—N1—C5A—C4A	179.0 (7)	C9—N2—C8—C7	−0.4 (4)
Co1—N3—C11—C12	173.84 (19)	C10—C6—C7—C8	0.0 (4)
Co1—N3—C15—C14	−173.5 (3)	C11—N3—C15—C14	3.8 (4)
Co1^iii^—N4—C18—C17	172.36 (19)	C11—C12—C13—C14	4.2 (4)
Co1^iii^—N4—C19—C20	−172.32 (19)	C11—C12—C13—C16	−175.8 (2)
Co2—O5—C35—O6	6.2 (4)	C12—C13—C14—C15	−4.0 (4)
Co2—O5—C35—C36	−173.12 (14)	C12—C13—C16—C17	−31.6 (3)
Co2—N2—C8—C7	177.9 (2)	C12—C13—C16—C20	149.3 (2)
Co2—N2—C9—C10	−177.8 (2)	C13—C14—C15—N3	0.0 (5)
Cl1—C24—C25—C26	179.2 (3)	C13—C16—C17—C18	−176.3 (2)
Cl2—C31—C32—C33	−179.1 (3)	C13—C16—C20—C19	176.0 (2)
Cl3—C38—C39—C40	178.5 (3)	C14—C13—C16—C17	148.4 (3)
O1—C21—C22—C23	5.0 (3)	C14—C13—C16—C20	−30.7 (4)
O1—C21—C22—C27	−178.3 (2)	C15—N3—C11—C12	−3.6 (4)
O2—C21—C22—C23	−172.9 (2)	C16—C13—C14—C15	176.0 (3)
O2—C21—C22—C27	3.8 (3)	C16—C17—C18—N4	−0.3 (4)
O3—C28—C29—C30	−2.4 (3)	C17—C16—C20—C19	−3.1 (4)
O3—C28—C29—C34	176.1 (2)	C18—N4—C19—C20	1.8 (4)
O4—C28—C29—C30	179.7 (2)	C19—N4—C18—C17	−2.1 (4)
O4—C28—C29—C34	−1.7 (4)	C20—C16—C17—C18	2.9 (4)
O5—C35—C36—C37	3.5 (3)	C21—C22—C23—C24	175.2 (2)
O5—C35—C36—C41	−176.3 (2)	C21—C22—C27—C26	−175.9 (3)
O6—C35—C36—C37	−175.9 (2)	C22—C23—C24—Cl1	−178.1 (2)
O6—C35—C36—C41	4.3 (4)	C22—C23—C24—C25	1.2 (5)
N1—C1—C2—C3	−2.6 (9)	C23—C22—C27—C26	0.8 (4)
N1—C1A—C2A—C3	3.5 (15)	C23—C24—C25—C26	−0.2 (5)
N2—C9—C10—C6	−0.4 (5)	C24—C25—C26—C27	−0.6 (5)
N3—C11—C12—C13	−0.4 (4)	C25—C26—C27—C22	0.2 (5)
N4—C19—C20—C16	0.8 (4)	C27—C22—C23—C24	−1.5 (4)
C1—N1—C5—C4	2.8 (8)	C28—C29—C30—C31	178.6 (2)
C1—C2—C3—C4	5.3 (7)	C28—C29—C34—C33	−178.2 (3)
C1—C2—C3—C6	−177.8 (4)	C29—C30—C31—Cl2	179.59 (19)
C2—C3—C6—C7	18.7 (6)	C29—C30—C31—C32	−1.1 (4)
C2—C3—C6—C10	−161.2 (6)	C30—C29—C34—C33	0.3 (4)
C4—C3—C6—C7	−164.7 (6)	C30—C31—C32—C33	1.6 (5)
C4—C3—C6—C10	15.5 (6)	C31—C32—C33—C34	−1.1 (5)
C5—N1—C1—C2	−1.6 (8)	C32—C33—C34—C29	0.2 (5)
C5—C4—C3—C2	−4.2 (8)	C34—C29—C30—C31	0.1 (4)
C5—C4—C3—C6	178.9 (5)	C35—C36—C37—C38	−179.6 (2)
C1A—N1—C5A—C4A	−3.0 (12)	C35—C36—C41—C40	179.6 (3)
C1A—C2A—C3—C4A	−6.2 (12)	C36—C37—C38—Cl3	−178.55 (19)
C1A—C2A—C3—C6	179.5 (7)	C36—C37—C38—C39	0.0 (4)
C2A—C3—C6—C7	−16.8 (11)	C37—C36—C41—C40	−0.2 (4)
C2A—C3—C6—C10	163.3 (11)	C37—C38—C39—C40	−0.1 (5)
C4A—C3—C6—C7	169.2 (8)	C38—C39—C40—C41	0.0 (5)
C4A—C3—C6—C10	−10.7 (9)	C39—C40—C41—C36	0.2 (5)
C5A—N1—C1A—C2A	1.2 (12)	C41—C36—C37—C38	0.1 (4)

Symmetry codes: (i) *x*−1, *y*, *z*; (ii) −*x*+1, −*y*+2, −*z*; (iii) *x*+1, *y*, *z*.

### Hydrogen-bond geometry (Å, º)

**Table:**

*D*—H···*A*	*D*—H	H···*A*	*D*···*A*	*D*—H···*A*
O7—H7*A*···O6^ii^	0.85 (2)	1.84 (2)	2.648 (3)	158 (3)
O7—H7*B*···O8^iv^	0.85 (2)	1.93 (2)	2.777 (10)	177 (3)
O7—H7*B*···O8*A*^iv^	0.85 (2)	1.88 (4)	2.72 (3)	168 (3)
O8—H8*A*···O4	0.82	1.90	2.708 (10)	169
O8*A*—H8*AA*···O4	0.82	2.04	2.67 (3)	133
C1—H1···O3	0.93	2.59	3.102 (7)	115
C5—H5···O1	0.93	2.48	3.057 (9)	121
C1*A*—H1*A*···O3	0.93	2.52	3.088 (9)	120
C5*A*—H5*A*···O1	0.93	2.33	2.991 (12)	128
C9—H9···O5	0.93	2.71	3.189 (3)	113
C11—H11···O2	0.93	2.57	3.084 (3)	115
C15—H15···N1	0.93	2.60	3.198 (4)	123
C26—H26···O6^v^	0.93	2.60	3.524 (4)	176

Symmetry codes: (ii) −*x*+1, −*y*+2, −*z*; (iv) *x*, *y*, *z*−1; (v) *x*, *y*−1, *z*+1.

## References

[bb1] Bruker (2016). *APEX3*, *SAINT* and *SADABS*. Bruker AXS Inc., Madison, Wisconsin, USA.

[bb2] Dey, D., Roy, S., Purkayastha, R. N. D., Pallepogu, R., Male, L. & Mckee, V. (2011). *J. Coord. Chem.* **64**, 1165–1176.

[bb3] Dolomanov, O. V., Bourhis, L. J., Gildea, R. J., Howard, J. A. K. & Puschmann, H. (2009). *J. Appl. Cryst.* **42**, 339–341.

[bb4] Fang, Z. & Nie, Q. (2011). *J. Coord. Chem.* **64**, 2573–2582.

[bb5] Fu, S.-J., Cheng, C.-Y. & Lin, K.-J. (2007). *Cryst. Growth Des.* **7**, 1381–1384.

[bb6] Islam, S., Datta, J., Maity, S., Dutta, B., Ahmed, F., Ghosh, P., Ray, P. P. & Mir, M. H. (2019). *ChemistrySelect*, **4**, 3294–3299.

[bb7] Kaes, C., Katz, A. & Hosseini, M. W. (2000). *Chem. Rev.* **100**, 3553–3590. 10.1021/cr990376z 11749322

[bb8] Kar, P., Biswas, R., Ida, Y., Ishida, T. & Ghosh, A. (2011). *Cryst. Growth Des.* **11**, 5305–5315.

[bb9] Kutasi, A. M., Batten, S. R., Harris, A. R., Moubaraki, B. & Murray, K. S. (2002). *CrystEngComm*, **4**, 202–204.

[bb10] Lin, R.-G. (2015). *Inorg. Chim. Acta*, **432**, 46–49.

[bb11] Madalan, A. M., Paraschiv, C., Sutter, J.-P., Schmidtmann, M., Müller, A. & Andruh, M. (2005). *Cryst. Growth Des.* **5**, 707–711.

[bb12] Piromchom, J., Wannarit, N., Boonmak, J., Pakawatchai, C. & Youngme, S. (2014). *Inorg. Chem. Commun.* **40**, 59–61.

[bb13] Saelim, T., Chainok, K., Kielar, F. & Wannarit, N. (2020). *Acta Cryst.* E**76**, 1302–1306. 10.1107/S2056989020009482 PMC7405572 32844018

[bb14] Seidel, R. W., Goddard, R., Zibrowius, B. & Oppel, I. M. (2011). *Polymers*, **3**, 1458–1474.

[bb15] Sheldrick, G. M. (2015*a*). *Acta Cryst.* A**71**, 3–8.

[bb16] Sheldrick, G. M. (2015*b*). *Acta Cryst.* C**71**, 3–8.

[bb17] Takahashi, K., Hoshino, N., Takeda, T., Noro, S., Nakamura, T., Takeda, S. & Akutagawa, T. (2014). *Dalton Trans.* **43**, 9081–9089. 10.1039/c4dt00258j 24804804

[bb18] Takahashi, K., Hoshino, N., Takeda, T., Noro, S., Nakamura, T., Takeda, S. & Akutagawa, T. (2015). *Inorg. Chem.* **54**, 9423–9431. 10.1021/acs.inorgchem.5b01168 26381225

[bb19] Topor, A., Avram, D., Dascalu, R., Maxim, C., Tiseanu, C. & Andruh, M. (2021). *Dalton Trans.* **50**, 9881–9890. 10.1039/d1dt01550h 34195749

[bb20] Xin-Jian, W., Yi-Ping, C., Ze-Min, X., Su-Zhi, G., Feng, C., Ling-Yan, Z. & Jian-Zhong, C. (2013). *J. Mol. Struct.* **1035**, 318–325.

