# Supplementary material for: Synthesis and crystal structure of poly[[bis­(aqua-κO)tetra­kis­(μ-4,4′-bi­pyridine-κ2 N:N′)hexa­kis­(3-chloro­benzoato)-κ5 O;κ2 O:O′-tricobalt(II)] methanol disolvate]
Source: Acta Crystallogr E Crystallogr Commun. 2022 Feb 1;78(Pt 3):255–8. doi: 10.1107/S2056989022000731 (PMC8900501; doi:10.1107/S2056989022000731)
Supplement: Supplementary file 3 [file e-78-00255-sup4.pdf]

## Supporting Information

### Synthesis and crystal structure of poly[[bis(aqua-*κO*)tetrakis(*μ*-4,4′-bipyridine)-$\kappa^2N$:$N'$)hexakis (3-chlorobenzoato)-$\kappa^5O$;$\kappa^2O$:$O'$-tricobalt(II)] methanol disolvate]

Phakamat Promwit[a], Kittipong Chainok[b] and Nanthawat Wannarit[a,b]*

**Experiment:**

All chemicals and solvents were of analytical grade purchased commercially and used without further purification. Elemental analyses (C, H and N) were performed on a Perkin-Elmer PE-2400 CHNS/O analyses. IR data were recorded on a Perkin Elmer infrared spectrophotometer with KBr pellets in the 400–4000 $cm^{-1}$ region. Powder X-ray diffraction pattern was obtained on PAN analytical Empyrean X-ray diffractometer in the 2theta range of 5-50° at room temperature and X-ray structure data analysis of the suitable single crystal was collected with a Bruker D8 Advance A25, using Mo-Kα radiation (λ = 0.71073 Å) at 296 K. Electronic spectra were recorded on Shimadzu UV-2600 UV-vis spectrophotometer in the range of 400-1100 $cm^{-1}$. TGA data was obtained on a TG-DTA 2010S MAC apparatus and Hitachi STA7200 thermal analyzer between 35-800°C in $N_2$ atmosphere with heating rate of 10 °C/min.

**Figure Caption**

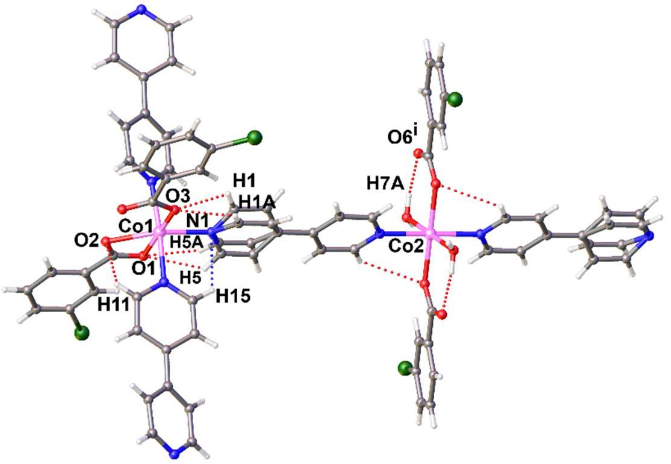


**Figure S1.** Representation of intramolecular hydrogen bonding interactions and hydrogen bonding between methanol lattice solvent and 3-Clbenz of the title compound. (Symmetry code (i) 1-x, 2-y, -z)

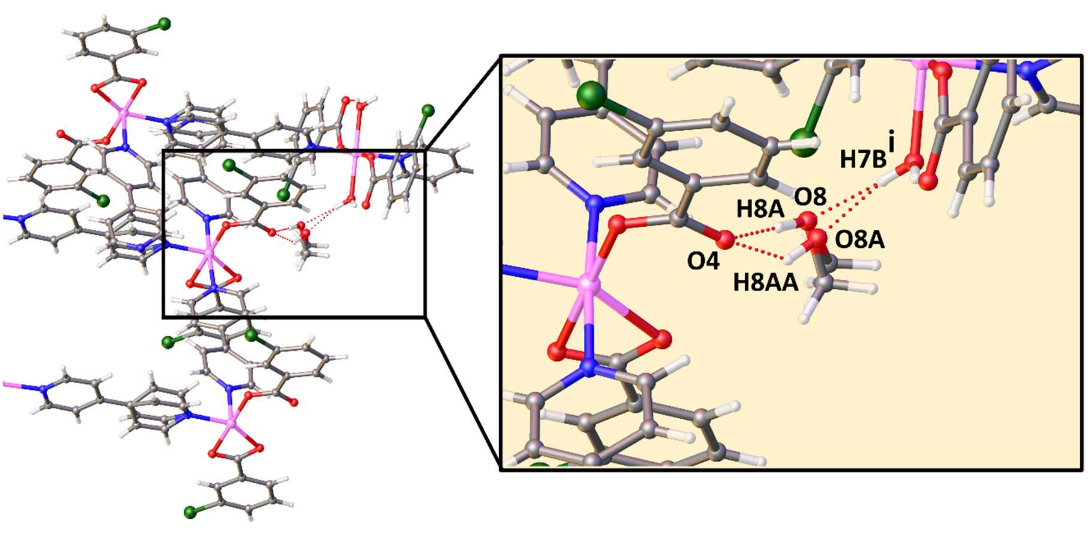


**Figure S2.** Representation of intermolecular hydrogen bonding interactions between methanol lattice solvent and the ladder chains (Symmetry code: (i) 1-x, 2-y, 1-z)

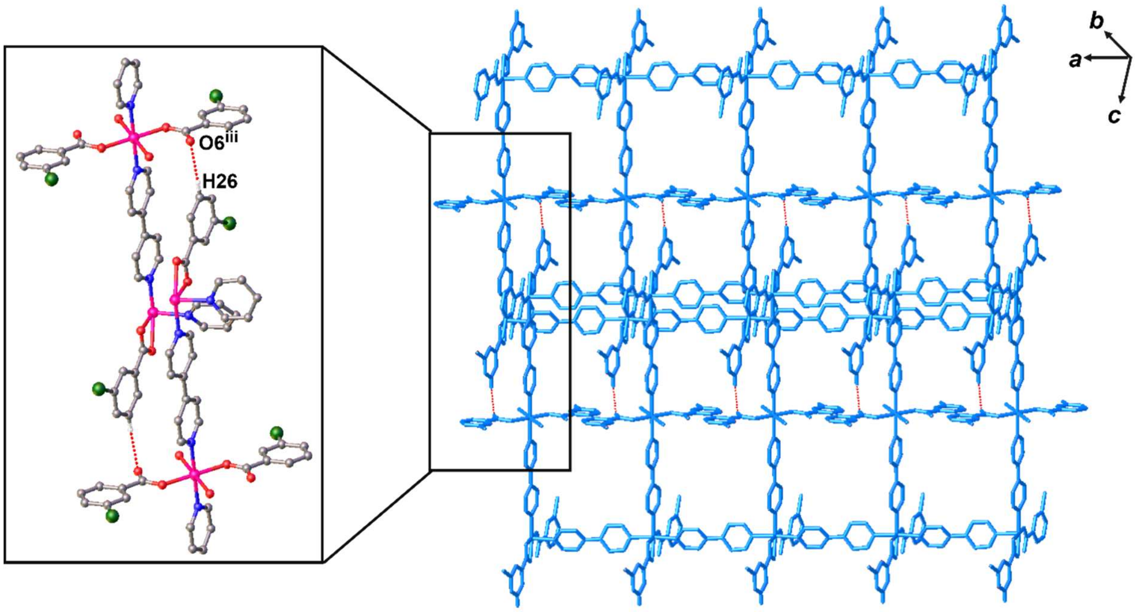


**Figure S3.** Representation of intermolecular hydrogen bonding interactions between adjacent ladder chains with C26-H26···O6$^{iii}$ (Symmetry code: (iii) *x*, *y*-1, *z*+1)

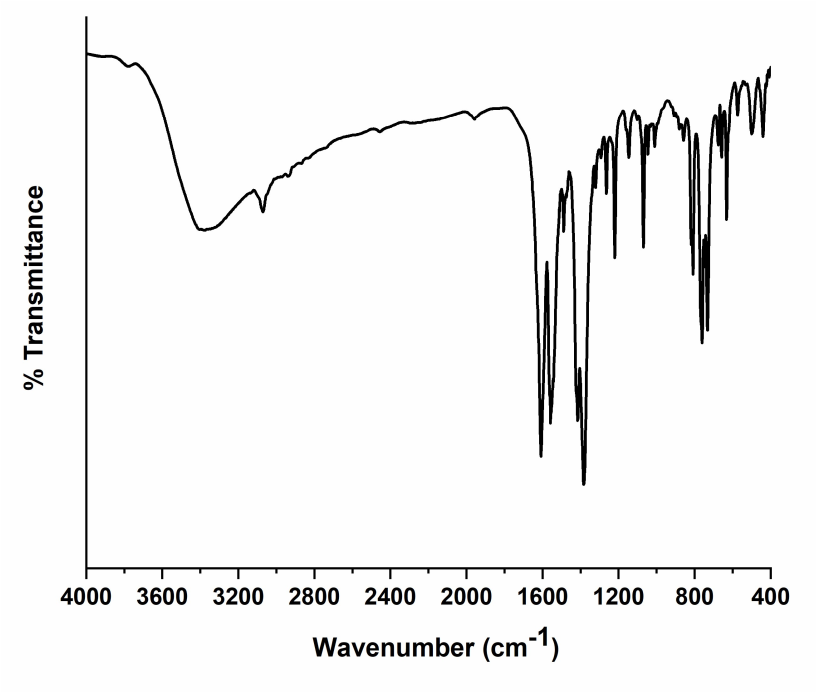


**Figure S4.** FT-IR spectrum of the title compound

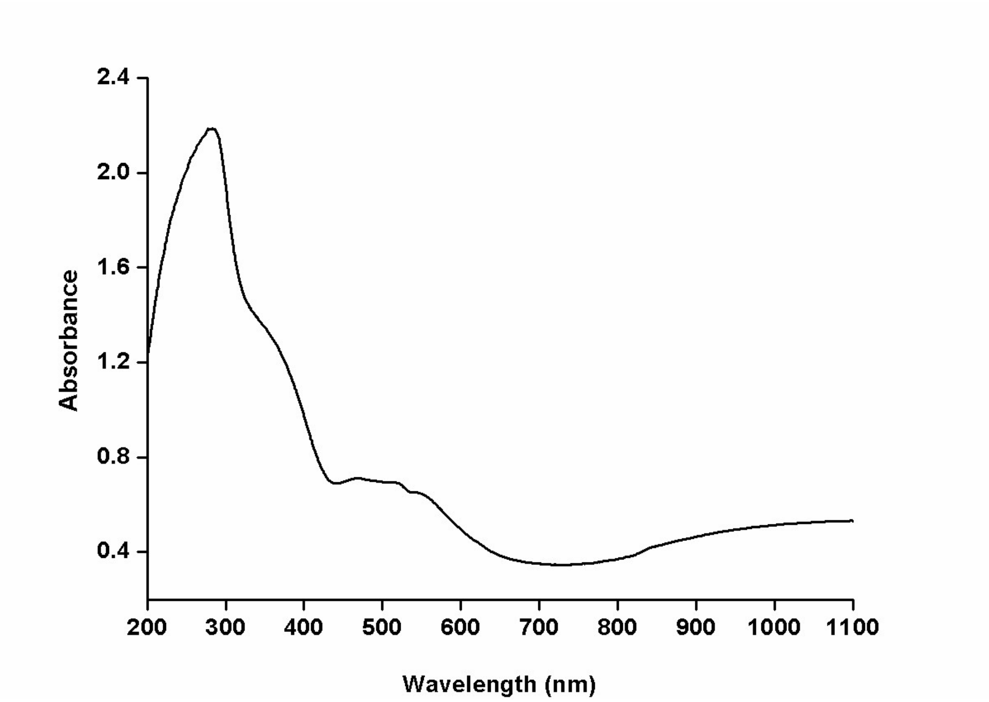


**Figure S5.** Solid state diffuse reflectance spectrum of the title compound

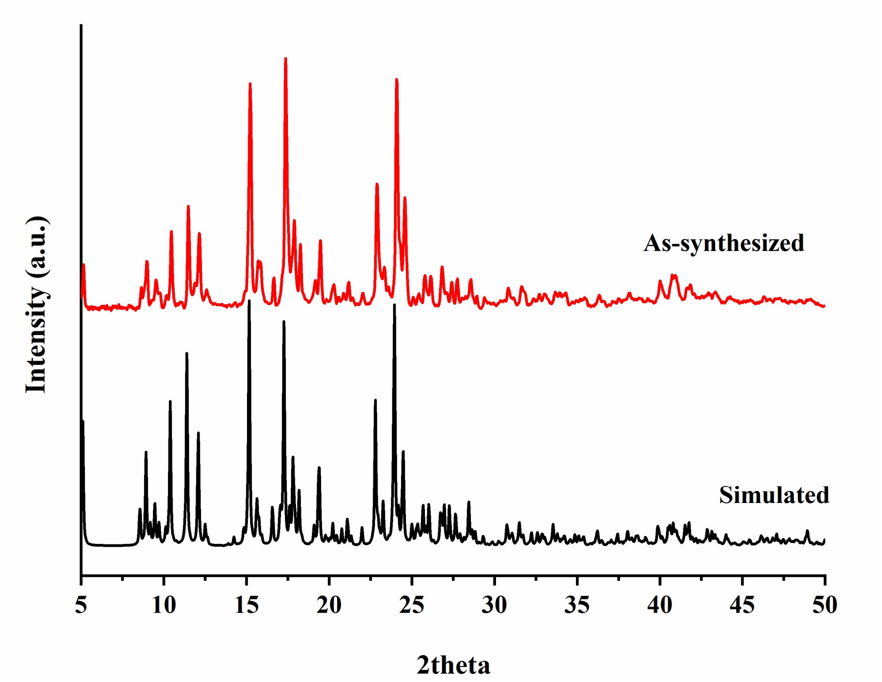


**Figure S6.** Powder XRD patterns of the title compound

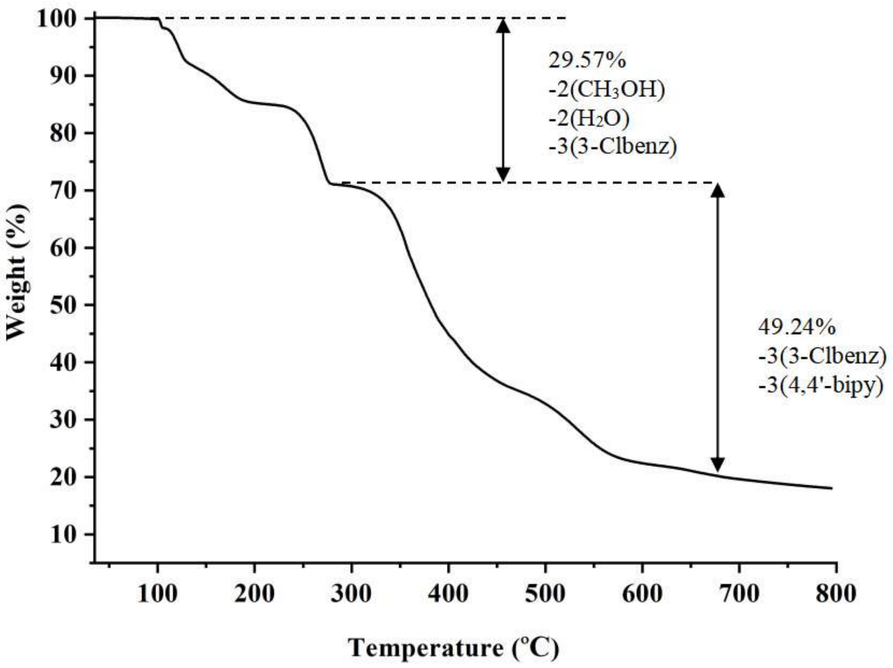


**Figure S7**. TGA curve of the title compound
